# Sac-Type Congenital Diaphragmatic Hernia: A Case Report of Two Siblings

**DOI:** 10.1155/2018/3270526

**Published:** 2018-08-12

**Authors:** Chisato Kodera, Takashi Ohba, Tomomi Hashimoto, Munekage Yamaguchi, Hidetaka Yoshimatsu, Hidetaka Katabuchi

**Affiliations:** ^1^Department of Obstetrics and Gynecology, Faculty of Life Science, Kumamoto University, Japan; ^2^Department of Obstetrics and Gynecology, Tokyo Women's Medical University Hospital, Japan; ^3^Department of Pediatrics, Faculty of Life Sciences, Kumamoto University, Japan

## Abstract

Congenital diaphragmatic hernia (CDH), a herniation of the abdominal contents through a defect or hypoplasia of the diaphragm, is a relatively common, severe congenital anomaly. Here we present the first case of two siblings with possibly isolated sac-type CDH and with a suspected genetic etiology. Although sibling recurrence of isolated CDH is rare, the incidence is higher than in the general population. Additionally, the second child had a more severe respiratory disorder than the first child. It is to be noted that siblings of children having isolated CDH are at risk for CDH, and prenatal evaluation should be considered individually.

## 1. Introduction

Congenital diaphragmatic hernia (CDH) is a herniation of the abdominal content through a defect or hypoplasia of the diaphragm and is associated with varying degrees of pulmonary hypoplasia. The severity of birth asphyxia varies from lethal to asymptomatic, and the overall neonatal mortality rate of prenatally diagnosed CDH has been reported to be 30%–60% despite optimal postnatal treatments [1].

The malformation commonly manifests as a hole or discontinuity in the diaphragm. Its exact etiology is unknown; however, it is now understood that the diaphragmatic defect develops owing to a failure of the pleuroperitoneal membranes to fuse during fetal development. In very few cases, the defect is not a hole but a thinning or incomplete muscularization of the diaphragm, which is generally referred to as sac-type CDH. Although CDH is usually a sporadic malformation, some sibling recurrences of isolated CDH have been documented [2–4]. Here, we present two siblings with isolated sac-type CDH who showed individual differences in symptom severity.

## 2. Case Presentation

**Figure 1:**
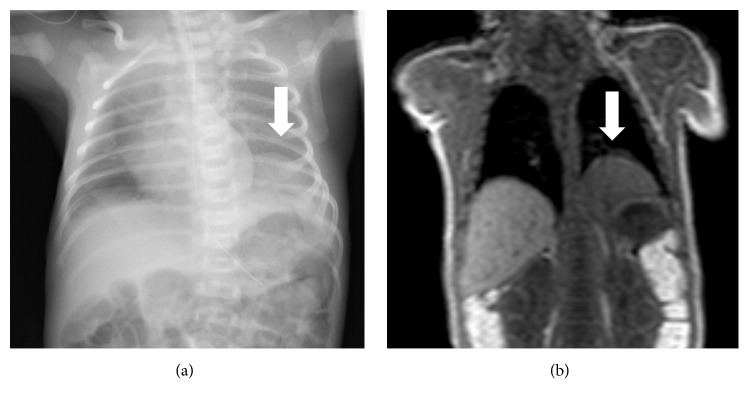
**(a)** A chest X-ray showed eventration of the left hemidiaphragm* (arrow)* on the first day after birth.** (b)** Magnetic resonance imaging revealed left sac-type CDH* (arrow)* at 21 days after birth.

A 28-year-old nulligravid Japanese woman was referred to Kumamoto University Hospital at 34 weeks of gestation because of symmetrical fetal growth restriction (FGR). In her family, there was no history of toxoplasmosis; rubella, cytomegalovirus, and herpes simplex virus infections; drug ingestion; consanguineous marriage; or genetic diseases. Her healthy partner had a familial trend of being small for gestational age (SGA) at birth. Cesarean section was performed at 37 weeks of gestation due to FGR and nonreassuring fetal status. A female infant weighing 1,498 g (−3.4 SD) was born with Apgar scores of 8 and 9 at 1 and 5 min, respectively. The newborn infant required 0.25–0.5 L/min nasal oxygen soon after birth, and her chest X-ray examination (Figure 1(a)) 1 day after birth revealed left CDH. Sac-type CDH was suspected on magnetic resonance imaging (MRI) at 21 days after birth (Figure 1(b)). Radical operation for CDH was performed at 30 days after birth, and the diagnosis of left sac-type CDH was confirmed. No associated abnormalities were detected. The postoperative course and subsequent development of the baby were uneventful except for insufficient postnatal catch-up growth.

**Figure 2:**
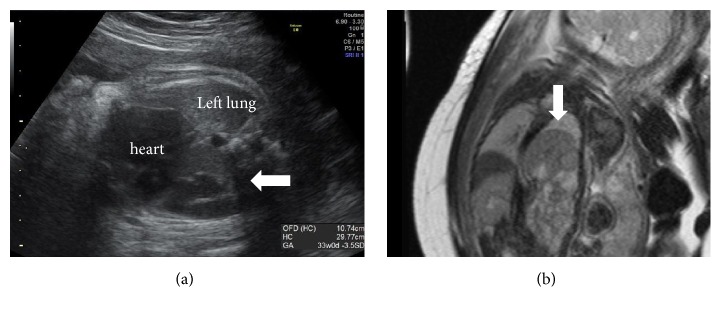
**(a)** Fetal echography showed a smooth-surface cystic lesion 29 × 44 mm in diameter in the left dorsal thorax* (arrow)*.** (b)** Magnetic resonance imaging at 37 weeks of gestation suggested left sac-type CDH. The stomach and spleen were herniated into the left chest* (arrow)*.

Following a miscarriage in the first trimester, the mother was referred to our hospital at 30 weeks of gestation for appropriate management of FGR 5 years after her first parturition. Obstetric sonography showed polyhydramnios and a simple, smooth cystic lesion in the left dorsal thorax, with the fetal heart displaced to the right side (Figure 2(a)). No associated malformations were detected. These findings suggested that the fetus had sac-type CDH. MRI revealed that the stomach and spleen were herniated into the sac-type CDH of the left chest (Figure 2(b)). The right lung–head ratio was 1.64, suggesting severe pulmonary hypoplasia after birth.

Elective cesarean section was performed at 38 weeks of gestation under general anesthesia. A male infant weighing 1,875 g (−3.5 SD) with an Apgar score of 1 at both 1 and 5 min was delivered. He was intubated immediately after birth, and oxygenation with intermittent positive-pressure ventilation was maintained. Furthermore, administration of catecholamine was required to maintain his blood pressure. On the first day after birth, surgical repair of CDH was performed. The left diaphragm was extended into the thorax, and the colon, spleen, and stomach were herniated into the sac. No associated abnormalities were detected. His postoperative course and subsequent development were also uneventful except for short stature. The parents did not wish to have chromosomal or genetic analysis performed on either sibling.

## 3. Discussion

CDH is a relatively common congenital anomaly that presents in 1 per 2,500–4,000 births. Its etiology is likely to be heterogeneous. Approximately 50%–60% of all CDHs exhibit isolated findings, whereas the remainder may be complex cases in which this anomaly forms as a part of genetic abnormality.

Complex CDHs, which account for approximately 5% of cases of familial CDH, are often associated with midline fusion defects such as neural tube defects, cleft lip and palate, and omphalocele, e.g., Donnai–Barrow syndrome inherited in an autosomal recessive (AR) manner [5], Matthew–Wood syndrome [6], and an autosomal dominant (AD) disorder with decreased penetrance and/or variable expressivity with GATA4 or ZEP4 mutation [7, 8]. Moreover, X-linked (XL) inheritance has also been reported [9]. The siblings in this study showed isolated sac-type CDH without other abnormal phenotypes, except FGR and SGA stature. XLR inheritance was unlikely in these siblings. AR or AD associated with incomplete penetrance and/or variable expressivity or germline mutation traits may have been the underlying genetic cause in our case.

**Figure 3:**
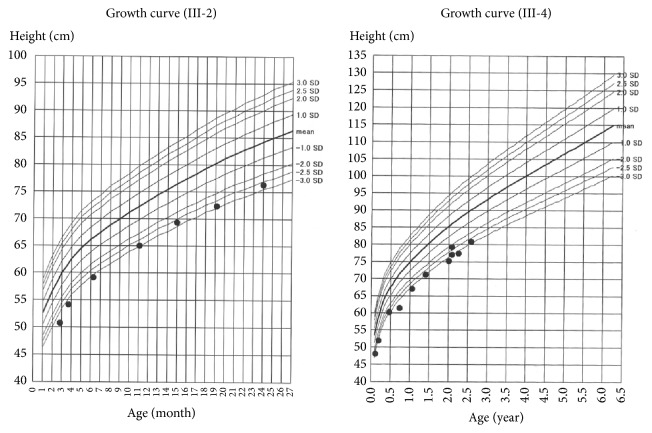
Growth curve of first (III-2) and second (III-4) infants suggested postnatal growth failure.

**Figure 4:**
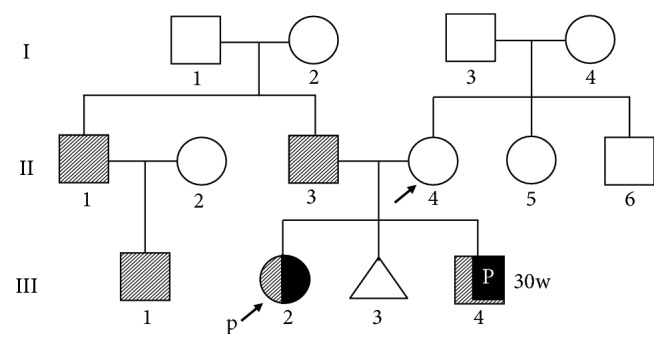
Pedigree of the family with familial CDH. Affected boy (■) and girl (●), spontaneous abortion (△), and SGA infant (lined square, lined circle) found in the paternal family.

Furthermore, both the siblings were associated with symmetrical FGR and postnatal short stature without postnatal catch-up growth (Figure 3). Their growth patterns were consistent with SGA stature. The findings suggested the possibility of a relevant part of the phenotype of syndromic disorder. SGA at birth was common in the paternal family (Figure 4). Although the father (II-3), uncle (II-1), and his son (III-1) showed short stature, there were no obvious associated malformations, including CDH. This suggested that sac-type CDH in these siblings was an isolated phenotype, but we could not rule out the possibility of a disorder including sac-type diaphragmatic hernia associated with SGA stature.

This is the first case report of familial sac-type CDH. It remains unclear whether common- and sac-type CDH develop via the same mechanism. In contrast to common-type CDH, sac-type CDH shows a convex, smooth surface of the herniated lesion and is more difficult to distinguish from the normal diaphragm on prenatal ultrasound examination. It has been reported that the presence of a hernia sac in CDH is associated with less visceral herniation, greater fetal lung growth, and better postnatal outcome [10, 11]. This is consistent with the clinical course of the elder child in our study. In contrast, the younger child required respiratory care immediately after birth, even with sac-type CDH. The present findings revealed that the phenotype of sac-type CDH may vary even among siblings. In addition, there may be affected individuals with subclinical diaphragmatic hernia in this family, which indicates the possibility of AD inheritance. Prenatal counseling and detailed ultrasound examinations for subsequent pregnancies should be offered to women with a family history of sac-type CDH.

In conclusion, we presented the first case of two siblings with possibly isolated sac-type CDH. Sac-type CDH is more difficult to diagnose prenatally and may vary in severity. Prenatal diagnosis with individual evaluation should be considered in subsequent pregnancies. SGA and short stature may be involved in the clinical etiology of sac-type CDH.
